# Role of CT angiography in detecting acute pulmonary embolism associated with COVID-19 pneumonia

**DOI:** 10.1007/s11547-021-01415-y

**Published:** 2021-09-17

**Authors:** Gabriele Masselli, Maria Almberger, Alessandra Tortora, Lucia Capoccia, Miriam Dolciami, Maria Rosaria D’Aprile, Cristina Valentini, Giacinta Avventurieri, Stefano Bracci, Paolo Ricci

**Affiliations:** 1 Unit of Emergency Radiology, Department of Radiological, Oncological and Pathological Sciences, Policlinico Umberto I, Sapienza University of Rome, Viale Regina Elena 324, 00161 Rome, Italy; 2 Unit of Radiology, Department of Radiological, Oncological, and Pathological Sciences, Policlinico Umberto I, Sapienza University of Rome, Rome, Italy

**Keywords:** COVID-19, Pulmonary embolism, ARDS, CT angiography

## Abstract

**Purpose:**

Recently coronavirus disease (COVID-19) caused a global pandemic, characterized by acute respiratory distress syndrome (ARDS). The aim of our study was to detect pulmonary embolism (PE) in patients with severe form of COVID-19 infection using pulmonary CT angiography, and its associations with clinical and laboratory parameters.

**Methods:**

From March to December 2020, we performed a prospective monocentric study collecting data from 374 consecutive patients with confirmed SARS-CoV-2 infection, using real-time reverse-transcriptase polymerase-chain-reaction (rRT-PCR) assay of nasopharyngeal swab specimens. We subsequently selected patients with at least two of the following inclusion criteria: (1) severe acute respiratory symptoms (such as dyspnea, persistent cough, fever > 37.5 °C, fatigue, etc.); (2) arterial oxygen saturation ≤ 93% at rest; (3) elevated D-dimer (≥ 500 ng/mL) and C-reactive protein levels (≥ 0.50 mg/dL); and (4) presence of comorbidities. A total of 63/374 (17%) patients met the inclusion criteria and underwent CT angiography during intravenous injection of iodinated contrast agent (Iomeprol 400 mgI/mL). Statistical analysis was performed using Wilcoxon rank-sum and Chi-square tests.

**Results:**

About, 26/60 patients (40%) were found positive for PE at chest CT angiography. In these patients, D-dimer and CRP values were significantly higher, while a reduction in SaO2 < 93% was more common than in patients without PE (*P* < 0.001). Median time between illness onset and CT scan was significantly longer (15 days; *P* < 0.001) in patients with PE. These were more likely to be admitted to the Intensive Care Unit (19/26 vs. 11/34 patients; *P* < 0.001) and required mechanical ventilation more frequently than those without PE (15/26 patients vs. 9/34 patients; *P* < 0.001). Vascular enlargement was significantly more frequent in patients with PE than in those without (*P* = 0.041).

**Conclusions:**

Our results pointed out that patients affected by severe clinical features of COVID-19 associated with comorbidities and significant increase of D-dimer levels developed acute mono- or bi-lateral pulmonary embolism in 40% of cases. Therefore, the use of CT angiography rather than non-contrast CT should be considered in these patients, allowing a better evaluation, that can help the management and improve the outcomes.

## Introduction

Since December 2019, a novel coronavirus (SARS-CoV-2) has spread rapidly from China to most countries causing a public health emergency, declared a pandemic in March 2020 [1].

As stated in the Chinese Center for Disease Control and Prevention report, the different forms of COVID-19 can be classified into mild, severe or critical based on the severity of the symptoms, with a frequency of approximately 81%, 14% and 5%, respectively [2, 3].

Furthermore, as several reports highlight [4–6], patients suffering from more severe forms of the disease have an increased risk of venous thrombosis and pulmonary embolism.

Thromboinflammation in COVID-19 manifests as elevated levels of procoagulants (such as von Willebrand factor) and endothelial dysfunction, which diminishes the protective antithrombotic activity of the endothelium, that leads to thrombosis, which can be a defense mechanism that compartmentalizes infection and prevents further dissemination [7, 8].

Given the large number of COVID-19 patients seeking medical care, the international society on thrombosis and hemostasis (ISTH) advocates the use of laboratory blood tests, including D-dimer test, prothrombin time and platelet count, to stratify the patients at risk of adverse outcome who need hospital admission [9].

Due to the primary involvement of the respiratory system, non-contrast chest CT has quickly proved to be a fundamental tool in the diagnosis, management and follow-up of COVID-19 patients [3, 10–12], also because of the increasing understanding of the temporal progression of the imaging findings at different stages of the disease [13].

However, despite the known increased risk of acute pulmonary embolism (APE) in patients with COVID-19 infection, only a few studies have evaluated the role of CT angiography in better understanding the disease [14–18].

Critically ill patients in the intensive care unit with COVID-19 have significantly higher rates of venous thromboembolism and thrombosis than COVID-19 patients on the wards [19, 20].

The aim of our study was to detect and assess pulmonary embolism in severely affected patients, using CT pulmonary angiogram, and its associations with clinical and laboratory parameters.

## Materials and methods

### Case selection

The institutional review board of our hospital approved the study.

From March 8th and December 2nd, 2020, we performed a prospective, single-center study collecting data from 374 consecutive patients admitted to our hospital with confirmed COVID-19 and who underwent non-contrast chest CT on admission.

A confirmed case was defined as positive by real-time reverse-transcriptase polymerase-chain-reaction (rRT-PCR) assay of nasopharyngeal swab specimens.

We selected patients confirmed to have SARS-CoV-2 infection and at least two of the following inclusion criteria: (1) severe acute respiratory symptoms (such as dyspnea, persistent cough, fever > 37.5 °C, fatigue, etc.); (2) arterial oxygen saturation ≤ 93% at rest; (3) elevated D-dimer (≥ 500 ng/mL) and C-reactive protein levels (≥ 0.50 mg/dL); (4) presence of comorbidities (such as hypertension, obesity, diabetes, ischemic heart disease, chronic pulmonary disease, cancer).

Patients without a confirmed SARS-CoV-2 infection were not eligible, because the initial level of COVID-19 suspicion varied considerably across these patients. We excluded patients with previous allergy to iodinated contrast agents, and those in whom no D-dimer testing had been performed during ED presentation.

A total of 63/374 (17%) patients met the inclusion criteria and underwent CT angiography.

### CT acquisition technique and imaging analysis

After unhanced CT chest scan, CT angiography was acquired on a 64 slice multi-detector CT (Siemens Somatom Sensation; Siemens Healthineers, Erlangen, Germany) with patient in supine position at maximum inspiration, after intravenous injection of 60 mL iodinated contrast agent (Iomeprol 400 mgI/mL, Bracco Imaging, Milan, IT) at a flow rate of 4 mL/s, triggered on the main pulmonary trunk. The scanning range was from the apex to lung base. CT scan settings were: 100 kV, 64 × 0,6 mm, rotation time 0.37 s, average tube current 207 mA, pitch 0.9 and CTDIvol 5.88 mGy. Images were all reconstructed with a slice-thickness of 1.00 mm, in both mediastinal and lung windows.

CT images were evaluated by two radiologists (G. M., M. A.) with, respectively 20 and 15 years of experience in chest imaging. Images were reviewed independently on Picture archiving and communication system (PACS) workstation (Carestream Health, Rochester, NY, USA) and in cases of discordance cases were discussed, and consensus reached.

CT studies that were limited by respiratory motion artifacts, or poor contrast opacification were excluded.

Each chest CT angiography was evaluated for the presence and location of pulmonary embolism, defined as major, lobar, segmental, or subsegmental pulmonary arteries based on the location of the most proximal luminal defect. The following CT features were also recorded: (a) ground-glass opacities (GGO), (b) consolidations (c) reticular pattern (defined as a diffuse interlobular septal thickening), (d) crazy paving pattern, (e) air bronchogram, (f) bronchiectasis, (g) pleural changes, (h) subpleural curvilinear changes, (i) fibrous stripes, (j) air bubble sign, (k) nodules, (l) halo sign, (m) reversed halo sign, (n) vascular enlargement (defined as a vessel diameter greater than 3 mm), (p) lymphadenopathy (defined as lymph node with short axis > 10 mm), (q) pleural and pericardial effusion.

We evaluated and compared the followings: (1) CT extent and distribution of pulmonary embolism; (2) need of recovery in critical care unit; (3) the presence of comorbidities; (4) the time delay from the onset of symptoms.

### Statistical analysis

Using PASS 11 program for sample size calculation, confidence level 95%, and a margin of error ± 0.1 and by reviewing previous study results by Léonard-Lorant et al. [15] showed the rate of pulmonary embolism among COVID-19-positive patients (30%); based on that, the required sample size will be 60 patients with COVID-19 to be sufficient to estimate the rate of pulmonary embolism in COVID-19 patients.

All the data were collected, compared, and analyzed using IBM SPSS statistics (V. 26.0, IBM Corp., USA, 2019) for data analysis. Data were expressed as mean ± SD for quantitative parametric measures in addition to median and percentiles for quantitative nonparametric measures and both number and percentage for categorized data. The following tests were done: 1) Comparison between two independent groups for nonparametric data using the Wilcoxon rank-sum test; 2) Chi-square test to study the association between every two variables or comparison between two independent groups as regards the categorized data. The probability of error at 0.05 was considered significant, while at 0.01 and 0.001 are highly significant.

## Results

### Patients data

A total of 63 out of 374 patients met the above inclusion criteria and underwent CT angiography. Three CT scans were excluded because they were affected by respiratory motion artifacts or poor opacification of the contrast medium, so the remaining 60 exams were admitted to our study.

Baseline characteristic of the population are shown in Table 1.

**Table 1:** Baseline population characteristics

	Patients with no PE at CT (*N* = 34; 57%)	Patients with PE at CT (*N* = 26; 43%)	*P* value
Age (years)	68 (57–78)	73 (67–82)	0.053
*Gender*			
Male	22 (67%)	20 (69%)	0.056
Female	12 (33%)	6 (31%)	0.067
Fever (> 37 °C)	28 (82%)	22 (84%)	0.083
Median value	37.9 °C	38.0 °C (37.5–38.5)	0.060
*Symptoms*			
Dyspnea	27 (81%)	23 (88%)	0.056
Cough	10 (29%)	7 (26%)	0.132
Fatigue	10 (29%)	8 (30%)	0.124
Tachycardia	6 (17%)	4 (15%)	0.145
Anosmia	2 (5%)	0 (0%)	0.078
Ageusia	1 (2.5%)	0 (0%)	0.085
Abdominal pain	1 (2.5%)	0 (0%)	0.085
*Comorbidities*			
COPD	5 (14%)	4 (15%)	0.074
Asthma	4 (12%)	4 (15%)	0.076
Emphysema	8 (24%)	7 (27%)	0.085
Cardiovascular	11 (32%)	9 (35%)	0.052
Hypertension	2 (6%)	0 (0%)	0.072
Diabetes	5 (15%)	3 (12%)	0.053
Chronic kidney	10 (29%)	9 (34%)	0.069
Cancer	4 (12%)	2 (8%)	0.054
Obesity	14 (41%)	10 (38%)	0.061
SaO_2_ (%) < 93%	12 (35%)	16 (62%)	**0.0008 **
D-Dimer (ng/mL)	4230 (1535–4473)	4473 (4382—4500)	**0.0007**
CRP (mg/dL)	13,20 (3,50–15,20)	13,50 (11,50—15,20)	**0.0002 **
Time from illness onset to CT angiography (days)	6 (4 – 13)	15 (8 – 18)	**0.0009 **
Invasive Mechanical Ventilation	9 (26%)	15 (57%)	**0.0007 **

Bold values are statistically significant (*P* < 0.05)

The overall median age was 68 years (57–78). 73% of patients were male (44/60). Fever (≥ 37.5 °C) was observed in 54/60 patients (90%). Dyspnea and cough were present in 56/60 (93%) and 17/60 (27%) patients, respectively. 51/60 (85%) patients presented comorbidities, with hypertension (33%), obesity (30%) and cardiovascular disease (24%) being the most common. D-Dimer and C-reactive protein (CRP) were increased in 94% and 97% of patients, with median value of 13.20 mg/dL (3.50–15.20) and 4230.00 ng/mL (1535.00–4473.00), respectively. 40% of patients (24/60) presented SaO2 < 93%. The median time from illness onset to CT angiography was eight days (5–17).

A total of 26/60 patients (40%) who underwent chest CT angiography were found positive for pulmonary embolism.

Among these, the location of the PE is as follows: 3 (5/26, 19%) central emboli, 12 (12/26, 46%) segmental embolus and 19 (19/26, 73%) subsegmental embolus. Eight (5/26, 19%) patients had a single embolus, while 21 (21/26, 81%) had multiple emboli.

In this group D-dimer and CRP values were significantly higher than in the general population and we observed a more frequent reduction in SaO2 (< 93%). Furthermore, median time between illness onset and CT angiography was significantly longer (15 days; 8–18).

**Fig. 1:**
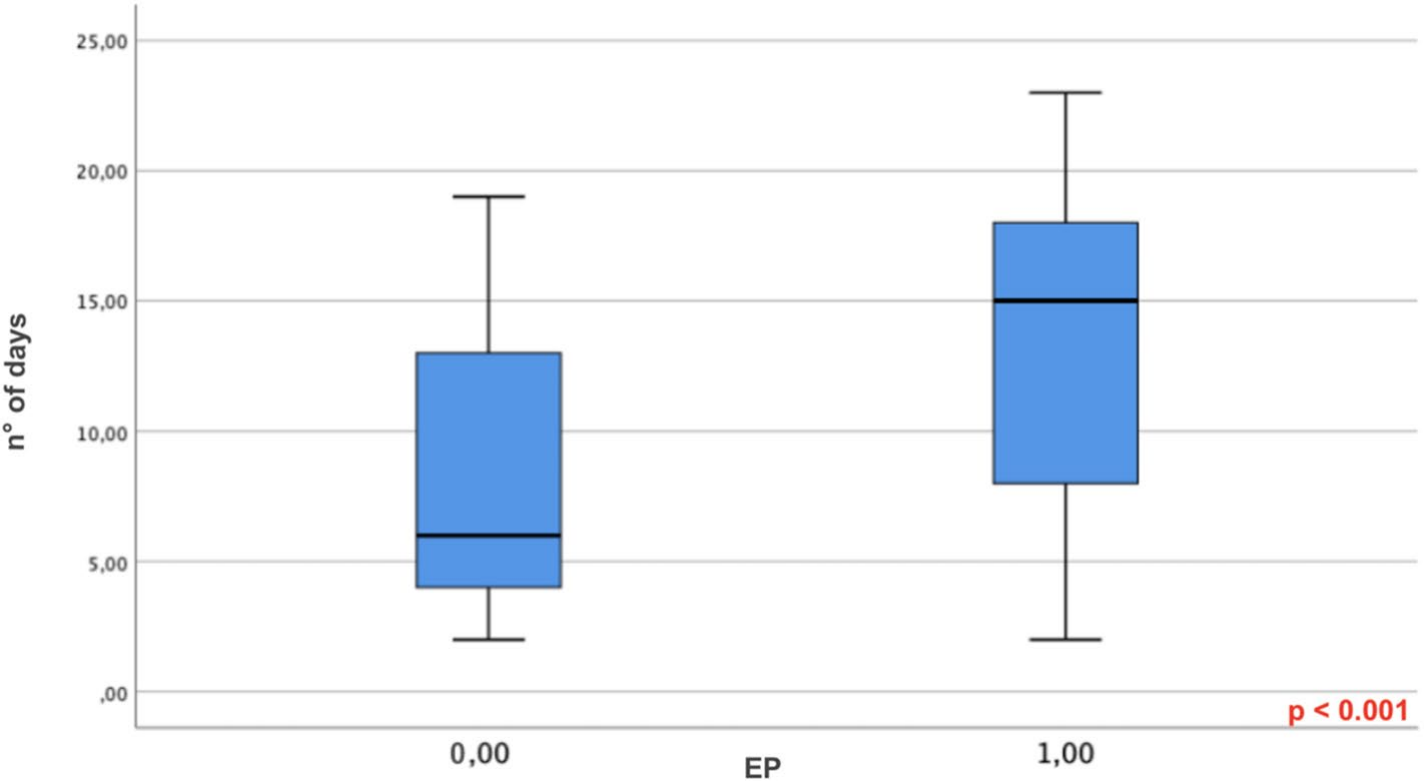
Time interval between symptoms onset and CT angiography in patient without (0, 00) and with pulmonary embolism (1, 00). Median value: 6 days versus 15 days (*P* < 0.001)

Patients with pulmonary embolism were more likely to be admitted to the Intensive Care Unit (19/26 [73%] vs. 11/34 [32%] patients, respectively; *P* < 0.001) and required mechanical ventilation more frequently than those without pulmonary embolism (15/26 patients [57%] vs. 9/34 patients [26%], *P* < 0.001) (Fig. 1).

### CT scan analysis

All 60 patients had signs of interstitial pneumonia, with different radiological patterns. The frequency and distribution of typical features is shown in Table 2.

**Table 2:** CT features

	Patients with no PE (*N* = 34)	Patients with PE (*N* = 26)	*P* value
Ground-glass opacities	17 (50%)	12 (46%)	0.081
Consolidations Interlobular septal thickening	20 (58%)	20 (76%)	0.125
Crazy paving pattern	15 (44%)	14 (50%)	0.099
Air bronchogram	9 (27%)	6 (23%)	0.074
Pleural changes	10 (30%)	9 (34%)	0.089
Subpleural curvilinear changes	11 (33%)	8 (31%)	0.068
Fibrous stripes	10 (30%)	8 (31%)	0.072
Air bubble sign	6 (18%)	4 (15%)	0,065
Nodules	4 (11%)	2 (7.5%)	0.055
Halo sign	3 (8.8%)	2 (7.5%)	0.091
Reversed halo sign	1 (2.5%)	0 (0%)	0.102
Vascular enlargement	0 (0%)	16 (61%)	**0.041**
Lymphadenopathy	11 (33%)	0 (0%)	0.052
Pleural and pericardial effusion	2 (5%)	2 (7.5%)	0.095
Multiple signs (> 2)	21 (62%)	18 (69%)	0.087

Bold value is statistically significant (*P* < 0.05)

**Fig. 2:**
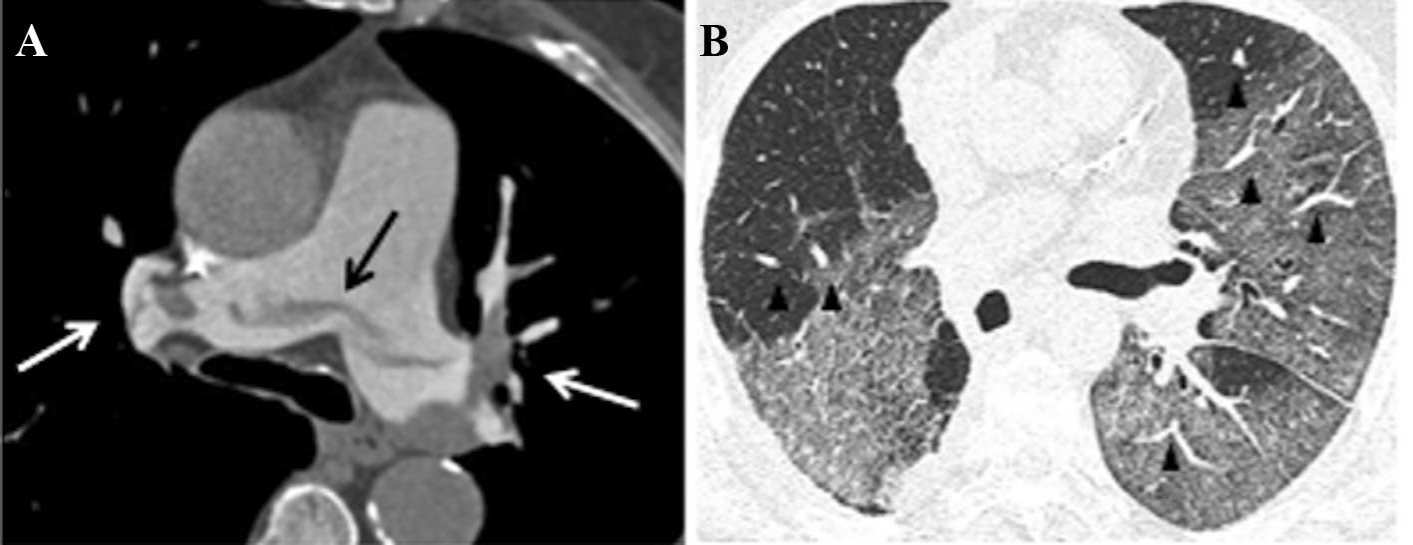
Pulmonary CT angiography of a 67-year-old male with acute pulmonary embolism. The CT scan was obtained six days after the onset of COVID-19 symptoms; on the same day the patient was transferred to the intensive care unit. **A,** Axial scan in mediastinal window shows linear saddle embolism of the pulmonary trunk (black arrow) and multiple bilateral filling defects involving lobar arterial branches (white arrows). **B,** Axial scans in lung window shows vascular enlargement (black arrowheads) and peripheral ground-glass opacities involving both lungs

All 40% of patients (26/60) had pulmonary embolism on CT angiography. The location of the most proximal luminal defect caused by pulmonary embolism was main pulmonary arteries in six cases (Fig. 2), lobar arteries in 12 cases, segmental or subsegmental arteries in seven cases and microembolism in just one case, with multiple locations in some patients.

**Fig. 3:**
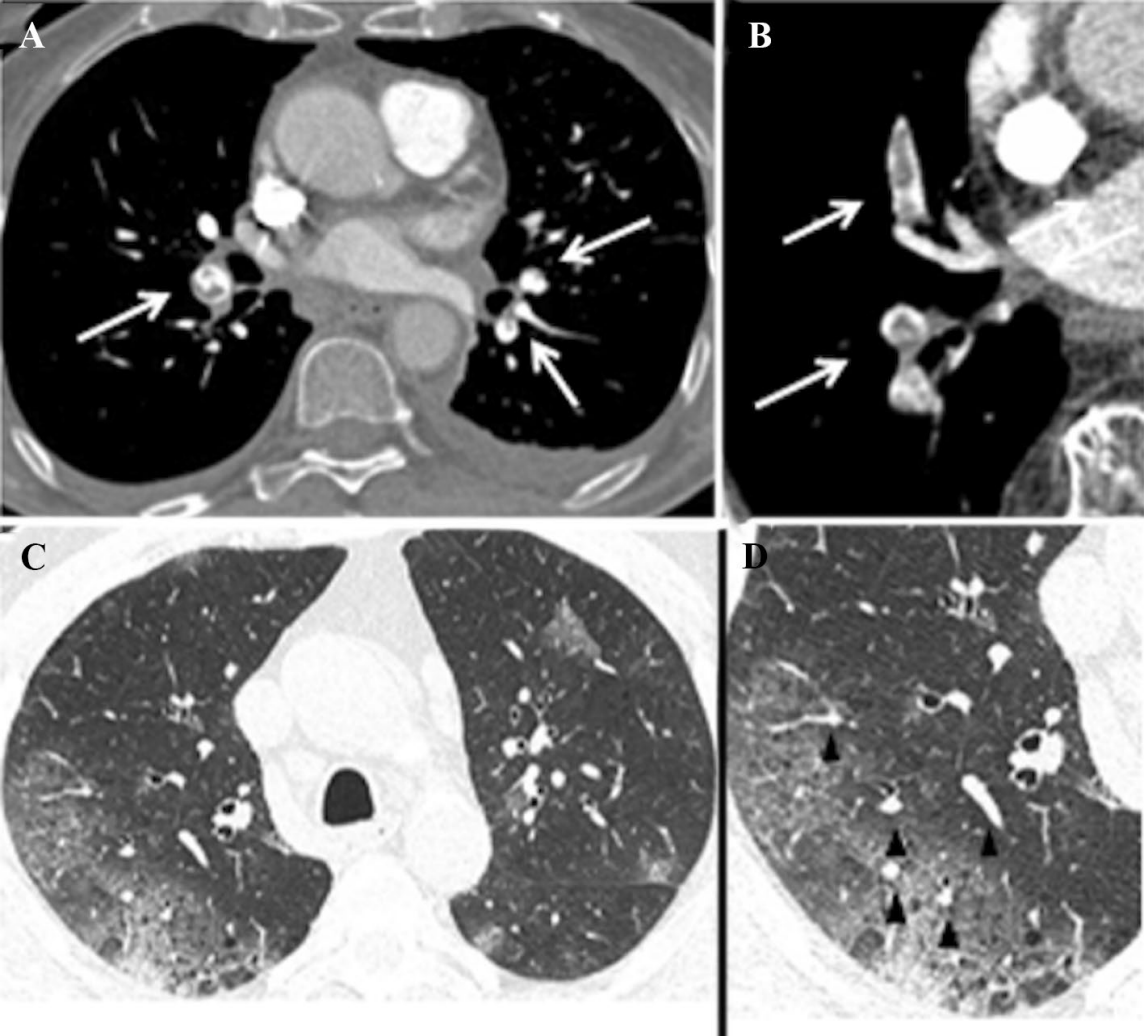
Pulmonary CT angiography of a 78-year-old male obtained 17 days after the onset of COVID-19 symptoms. **A**, **B** Axial contrast-enhanced CT scan shows bilateral filling defects involving lobar branches of the pulmonary artery (white arrows). **C**, **D,** Axial CT images (lung window) show peripheral ground-glass opacities with associated areas of consolidation, lung architectural distortion and vascular enlargement (black arrowheads)

Patients with pulmonary embolism had a significant increase in the frequency of the radiological sign of “vascular enlargement” (Fig. 3). In particular, we found a significant correlation in patients with main pulmonary arteries embolism (*P* = 0.010) or with microembolism (*P* = 0.032). Consolidations were significantly more frequent in patients with lobar embolism (*P* = 0.012). We found no significant correlation with the remaining imaging features.

Furthermore, in patients with main artery embolism, lobar embolism and microembolism we found a significant correlation with the presence of asthma (*P* < 0.007). Emphysema was significantly more frequent in patients with main artery embolism and lobar embolism (*P* < 0.0105).

## Discussion

In this study of 60 patients hospitalized with COVID-19 and suspected of PE, we found pulmonary embolism in 40%. Patients with pulmonary embolism were more likely to be admitted to the Intensive Care Unit (19/26 [73%] vs. 11/34 [32%] patients, respectively; *P* < 0.001) and required mechanical ventilation more frequently than those without pulmonary embolism (15/26 patients [57%] vs. 9/34 patients [26%], *P* < 0.001).

On univariate analysis, the only imaging characteristics on chest CT associated with APE was the “vascular enlargement”.

Current guidelines recommend performing non-contrast chest CT to assess the COVID-19 CT pattern and its extension.

The impact of thrombotic complications has been increasingly recognized as an important component of this disease and a subgroup of patients will develop severe respiratory distress syndrome, sepsis, septic shock and pulmonary embolism and require mechanical ventilation and recovery in the critical care unit more likely than the patients without pulmonary embolism [21].

Coagulopathy commonly occurs in sepsis and may predict outcomes in severe COVID-19 [22]. Prior reports suggest dysregulated hemostatic pathways and coagulation defects are frequently associated with COVID-19 infection; furthermore, inflammation of lung parenchymal and vascular endothelial cells may increase the release of procoagulant factors with higher risk for developing pulmonary embolism. Han et al. reported disturbed coagulation function in patients infected with SARS-CoV-2 as compared to healthy controls, including elevated D-dimer, fibrin/fibrinogen degradation products, and fibrinogen levels [23]. Additionally, two different studies by Zhou et al. [24] and Tang et al. [25] recently reported a positive correlation between elevated D-dimer levels on admission and in-hospital COVID-19 mortality, raising questions regarding potentially unknown pulmonary embolism and outlining the possible role of CT pulmonary angiography in patients affected by COVID-19 who develop a rapid clinical worsening.

Therefore, we performed contrast-enhanced CT for COVID-19 patients with severe clinical features to evaluate the lung parenchyma as well as other complications that may result in respiratory distress.

At multivariable analysis, pulmonary embolus was associated with invasive mechanical ventilation. Interestingly, extent of lesions was not associated with pulmonary embolus.

There has been association of better outcomes of COVID+ patients treated with anticoagulation prophylaxis, such as low-molecular-weight heparin in sepsis induced coagulopathy and high D-dimers. The identification of thromboembolic complications such as pulmonary embolism may aid in improving outcomes as diagnosed patients would be treated with anticoagulation.

The median time to develop PE was found to be 15 days. This is similar to Grillet et al. [16] who diagnosed PE at a mean of 12 days from the onset of the symptoms. The duration of illness in our opinion could raise a flag to continuous follow-up of other alarming symptoms and serial measures of D-dimer. We found a difference of median time from illness onset to CT angiography between PE and no PE. A possible explanation is that the difference in prevalence of disease could be related to the time between illness of disease and the CT angiography.

The radiological features of COVID-19 pneumonia does not allow for the selection of patients at risk of APE; however, in our study we found that vascular dilatation was seen in most of the cases with pulmonary embolism, which might be attributed to the swelling of the capillary wall caused by pro-inflammatory factors [26].

The presence of vascular dilatation described as the dilatation of pulmonary vessels around and within the lesions on CT no contrast images [27], could suggest performing CT pulmonary angiogram for ruling out the APE in COVID-19 patients.

We found a significantly higher D-dimer level in the APE group compared with the non-APE group. The D-dimer level seems to be an important parameter in the management of COVID-19 patients, making it possible both to assess the severity of the disease [24] and to suspect APE. Given that the increase in the D-dimer level can be linked to COVID-19, it would be interesting to evaluate on the basis of large-scale studies if there is a cut-off D-dimer level at which CTPA could be recommended to search for APE in COVID-19 patients [15].

Pending further data on D-dimer levels, we believe that all patients with COVID-19 pneumonia and an increased level of D-dimer should benefit from CTPA to eliminate APE, whenever possible.

While this study confirms several important clinical observations regarding thromboembolism and COVID-19, key practical questions remain unanswered. First is how to best identify patients with COVID-19 who will benefit from pulmonary CTPA. This is particularly difficult to determine. A study reporting low rates of venous thromboembolism during the 90 days following hospitalization for COVID-19 suggests that the incidence of untreated PE among COVID-19 may be small [28].

Second when pulmonary emboli are detected by CTPA, their clinical significance in the setting of COVID-19 may be ambiguous.

Limitations of our study included a relatively small sample size, and restriction to a single-health system.

In addition, as mentioned previously, CT angiography was not performed routinely for the initial diagnosis of COVID-19, and our patient population may have reflected those with more severe lung involvement.

In conclusion our results pointed out that patients affected by severe clinical features of COVID-19 associated with comorbidities and significant increase of D-dimer levels develop acute mono- or bi-lateral pulmonary in 40% of cases.

According to our observations this might especially be justified if non-enhanced CT shows a “vascular enlargement”, as this was the only imaging characteristic significantly associated with APE in our study.

Contrast-enhanced CT may be considered for these patients, allowing a better evaluation that can help the management.

## Data Availability

The datasets analyzed during this study are available from the corresponding author upon reasonable request.
